# The outcomes of dexmedetomidine and calcitriol on flap viability ^1^

**DOI:** 10.1590/s0102-865020200090000003

**Published:** 2020-09-30

**Authors:** Mustafa Sırrı Kotanoğlu, Aylin Akbulut, Koray Gürsoy, Gökhan Koca, Namık Özcan, Nihat Yumuşak, Mehmet Şenes, Gül Kırtıl, Meliha Korkmaz

**Affiliations:** I MD, Department of Anesthesiology and Reanimation, University of Health Sciences, Ankara Training and Research Hospital, Ankara, Turkey. Substantive scientific and intellectual contributions to the study.; II MD, Department of Nuclear Medicine, University of Health Sciences, Ankara Training and Research Hospital, Ankara, Turkey. Conception and design of the study, analysis and interpretation of data.; III MD, Associate Professor, Department of Plastic, Reconstructive and Aesthetic Surgery, University of Health Sciences, Ankara Training and Research Hospital, Ankara, Turkey. Technical procedures.; IV Associate Professor, Department of Nuclear Medicine, University of Health Sciences, Ankara Training and Research Hospital, Ankara, Turkey. Conception and design of the study, analysis and interpretation of data.; V Associate Professor, Department of Anesthesiology and Reanimation, University of Health Sciences, City Hospital, Ankara, Turkey. Substantive scientific and intellectual contributions to the study.; VI Associate Professor, Department of Pathology, Harran University Faculty of Veterinary Medicine, Sanliurfa, Turkey. Histopathological examinations.; VII Associate Professor, Department of Biochemistry, University of Health Sciences, Ankara Training and Research Hospital, Ankara, Turkey. Analysis and interpretation of data.; VIII MD, Department of Biochemistry, University of Health Sciences, Ankara Training and Research Hospital, Ankara, Turkey. Analysis and interpretation of data.; IX MD, PhD, Department of Anesthesiology and Reanimation, University of Health Sciences, Ankara Training and Research Hospital, Ankara, Turkey. Critical revision, final approval.

**Keywords:** Ischemia, Reperfusion Injury, Dexmedetomidine, Calcitriol, Rats

## Abstract

**Purpose:**

To evaluate protective effects of dexmedetomidine, calcitriol and their combination.

**Methods:**

Forty Wistar-albino rats were divided into 4 groups; group of Sham (Group Sham); group of dexmedetomidine (Group DEX); group of calcitriol (Group CAL) and group of dexmedetomidineandcalcitriol (Group DEX-CAL). Photographic analysis was used for macroscopic analysis and perfusion analyses were evaluated by scintigraphy. Additionally, tissue malondialdehyde (MDA) and total oxidant status (TOS) and total antioxidant activity (TAS) were recorded and oxidative stress index (OSI) was calculated. Each flap was assessed by histopathology.

**Results:**

Compared to Group Sham, the viable flap areas were higher in all treatment groups both by photographic image analyses and perfusion analyses (p<0.05). Group DEX-CAL had the highest viable flap percentage both in scintigraphic and photographic analyses; whereas Group Sham had the lowest viable flap percentage. Similarly, TAS and MDA levels were elevated and TOS levels were declined in all treatment groups compared to Group Sham (p<0.005). Histopathological analysis at flap demarcation zone confirmed neovascularization was significantly higher and edema, necrosis and inflammation were significantly lower in all treatment groups compared to Group Sham.

**Conclusion:**

The outcomes show that additional premedication with either dexmedetomidine or calcitriol or their combination reduces ischemia-reperfusion injury of flap area and show significant increase in the percentage of viable flap tissue.

## Introduction

Random pattern skin flaps are commonly indicated as a first-line treatment modality for skin defect reconstruction due to various reasons such as trauma, surgery and malformations. The most common complication of random skin flaps is the tissue necrosis, prominent in the distal portion of the flap mainly due to ischemia-reperfusion (IR) injury leading to partial flap loss. Because of the increasing popularity of flap surgery, a rising number of studies with several mediators have been on trial to prevent IR injury and consequently to improve the survival rate of flaps. To prevent partial flap loss, an ideal agent should have tissue-protective effects with anti-inflammatory and antioxidant properties but without side effects.

Dexmedetomidine is a highly selective alpha-2 adrenergic agonist causing sympatholysis and is widely used for sedation and analgesia without respiratory depression ^1^ . The U.S Food and Drug Administration approved DEX in 1999 for sedation of patients hospitalized in intensive care settings for regional ^2^ and general anesthesia ^3^ . The flap surgery procedures are commonly performed under general or regional anesthesia in humans; the effects of anesthetics on flap tissue are of clinical interest. In addition to its analgesic and sedative effects, dexmedetomidine has an anti-inflammatory effect through the cholinergic anti-inflammatory pathway which improves survival in experimental endotoxemia by inhibiting the inflammatory cytokines release ^4^ . Furthermore, the protective effect of dexmedetomidine to many organs such as heart, brain, kidney, liver and testis has been demonstrated by enhancing the vagus nerve excitability and producing hemodynamic stability ^5^ ^,^ ^6^ . Recently, the protective effect of dexmedetomidine preconditioning on IR injury has been shown in heart and in testis tissue experimental models ^7^ ^,^ ^8^ . A recent study reported that dexmedetomidine increases flap viability in the inferior epigastric island flaps ^9^ . A myocutaneous flap study utilizing dexmedetomidine for postoperative sedation showed that dexmedetomidine does not interfere with local perfusion or tissue metabolism in denervated musculocutaneous flaps ^10^ . Furthermore, a study on human endothelial cells has shown that dexmedetomidine can be safely used for long term sedation in patients receiving therapeutic angiogenesis for ischemic vascular disease ^11^ .

Calcitriol is a metabolite of vitamin D, also known as 1,25-dihydroxy vitamin D3, currently a commonly available agent on clinical osteoporosis. Furthermore, it has anti-inflammatory ^12^ and antioxidant ^13^ properties and also it promotes vascular endothelial growth factor (VEGF) expression ^14^ which may have a potential effect on the flap viability.

In the ischemic necrosis of skin flaps, both the adrenergic vasoconstriction and platelet aggregation in the microvascular system have major importance. After ischemia, Thromboxane A2 (TXA2) released from the platelets causes vasoconstriction and platelet aggregation. Endothelial cell migration mediated by TxA2 is stimulated by VEGF and basic fibroblast growth factor (bFGF). However, due to vascular damage, the prostacyclin from the vascular endothelium cannot be released to block the effect of TXA2 and eventually as the TXA2 increases and prostacyclin decreases, therefore, the perfusion of the tissue decreases ^15^ . In skin flaps, VEGF is secreted by keratinocytes and fibroblasts in dermis and epidermis, and in dermal vessels ^16^ and the vascularization of random skin flaps can be encouraged by the administration of VEGF ^17^ . However, calcitriol up-regulates VEGF levels in dermis and contributes vascularization in ischemic tissue ^14^ ^,^ ^18^ and also dexmedetomidine increases the production of VEGF ^19^ . Thus, by this mechanism, the combination of calcitriol and dexmedetomidine may potentially have a supporting effect on flap studies.

Based on previous encouraging results of calcitriol and dexmedetomidine, this study was designed to assess the protective effects of dexmedetomidine and also possible and/or additive effects of calcitriol on random skin flap survival which has not been evaluated through the literature. To evaluate the antioxidative properties of calcitriol and dexmedetomidine we have measured the tissue oxidant parameters, such as total oxidative status (TOS), and antioxidant parameters such as total antioxidant capacity (TAS) and Malondialdehyde (MDA) which is a lipid peroxidation product, commonly used for evaluation of tissue damage caused by the free radicals during reperfusion ^20^ . Additionally, we examined histopathological alterations in the flap tissue and macroscopic evaluation via photographic analysis; the perfusion of the flaps was evaluated via scintigraphic methods.

## Methods

The approval of the local ethical committee was received for this research and all the procedures were accordant with international health and medical research guidelines for animal welfare.

Six-month-old Wistar-albino male rats weighing 300-350 g were used in the study. The rats were accommodated in individual cages in an environmentally controlled animal room (temperature 22°C, humidity 40%–70%) on a 12-h light/dark cycle and fed with standard rat chow and tap water *ad libitum* . All interventional procedures and imaging studies were performed under general anesthesia with an intraperitoneal injection of 10 mg/kg of 2% xylazine (Rompun, Bayer, Leverkusen, Germany) and 75−100 mg/kg of ketamine hydrochloride (Ketalar, Eczacibasi, Istanbul, Turkey).

### Experimental protocol

The surgical procedures were performed under sterile conditions by a single plastic surgeon. After the removal of dorsal hair with an electric shaver, the modified version of the McFarlane flap was prepared in all rat groups. Caudal based skin flap (9×3 cm) was marked on the dorsum of the rat, beginning from the line connecting the iliac spines. To maintain a random pattern of blood circulation, the deep circumflex iliac artery and perforator vessels were cauterized and the flap was elevated below panniculus carnosus muscle (PCM) in all groups. The flaps were sutured back to their original position after 5 minutes without carrying out any procedure. Rats were randomized into 4 groups of 10 each. The first group was the Group Sham and received i.p. saline injections starting from 2 hours before flap elevation and continued daily for 7 days. Group DEX received 10 μg/kg i.p. dexmedetomidine ^9^ (Hipnodex, Haver Pharma Ilac A.S.) 30 minutes before McFarlane flap elevation. Group CAL received 2 μg/kg/day (Calcijex, Abbott, Turkey) i.p. calcitriol starting from 2 hours before McFarlane flap elevation and continued for consecutive 7 days, and Group DEX-CAL received i.p. 2 μg/kg/day calcitriol ^18^ 2 hours before McFarlane flap elevation and 10 μg/kg i.p. dexmedetomidine 30 minutes before McFarlane flap elevation and calcitriol was continued for the next 6 days.

### Clinical and photographic analysis

**Figure 1:**
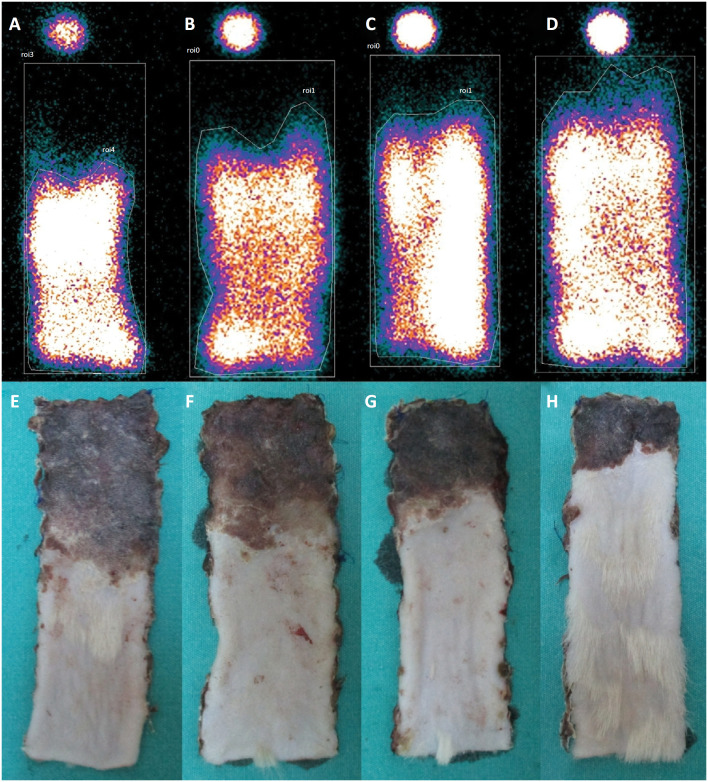
The scintigraphic perfusion images of the flaps of **a)** Group Sham, **b)** Group DEX, **c)** Group CAL, **d)** Group DEX-CAL and the corresponding macroscopic images, **e)** Group Sham, **f)** Group DEX, **g)** Group CAL, and **h)** Group DEX-CAL.

One week after the flap surgery, digital photographs of the flaps were taken from a distance of 50cm. The flap areas and the viable parts were calculated in mm2 by Digimizer image analysis software (Med-Calc Software, Ostend, Belgium) ( Fig. 1 ). The viable part of the flap was found by subtracting the necrotic part of the flap from the whole flap area.

### Radionuclide scintigraphic analysis

One week after the flap surgery, the rats underwent scintigraphic imaging at a gamma camera with a pinhole collimator (Siemens e.Cam, Siemens Medical Solutions, USA) in prone position after the injection with 1 milicurie of technetium-99m pertechnetate (Tc99m-PO4) in 0.1 mL of isotonic saline via the tail vein. Dynamic images were obtained simultaneously and blood pool images were acquired 5 minutes after injection in a 256 × 256 pixel matrix and the rats were then sacrificed and the flap tissue was removed. The flap tissue only image was acquired for 5 minutes to prevent the background activity scattering from the organs of the rat. The distal border of the flap tissue was marked with a Tc99m-PO4-soaked cotton marker. The interpretation of the scintigraphic analysis was assessed with two-experienced nuclear medicine physicians blinded to the rat groups. The scintigraphic images of the viable parts of the flaps were drawn manually, and a rectangular region of interest (ROI) was drawn encompassing the whole flap.

### Histopathological assessment

The sacrification of the rats was acquired with high-dose ketamine after scintigraphic imaging. The skin flaps were fixed in 10% buffered formaldehyde and embedded in paraffin blocks. After paraffin embedding, the pathologist divided the flap area into 3 regions the distal part as the necrotic zone, the demarcation zone, which has both viable and necrotic areas, and the proximal part as the pedicle zone. The demarcation zone including both viable and necrotic zones thorough 2 cm widths of the flaps were taken into account. Then 5-mm sections were obtained, deparaffinized, stained with hematoxylin-eosin, and examined under a light microscope by an experienced veterinary pathologist in a randomly numbered blind fashion. All the zones were scored according to edema, inflammation, necrosis parameters from 0 to 4: whereas score 0 is none, score 1is mild, score 2 is positive, score 3 is strongly positive, score 4 is severe positive. Also, the number of mature vessels containing erythrocytes for 10 most intense vascularized fields were counted and the average of these fields was calculated.

### Biochemical analysis

Tissue samples were weighed and homogenized with an automatic homogenizer (Heidolph DIAX 900) in cold phosphate buffer saline (PBS; 50 mM, pH 7.4) at a ratio of 1/10. The homogenates were centrifuged for 10 minutes at 10.000 g and supernatants were used for biochemical analysis.

Total antioxidant status (TAS), was measured with the spectrophotometric method developed by Erel, using Rel Assay brand commercial kits (Rel Assay Kit Diagnostics, Turkey). Trolox, a water-soluble analogue of vitamin E, was used as calibrator ^21^ . Total oxidant status (TOS) was measured with the spectrophotometric method developed by Erel, using Rel Assay brand commercial kits (Rel Assay Kit Diagnostics, Turkey). Hydrogen peroxide was used as calibrator ^22^ . The results are expressed in μmol H2O2equiv./L. OSI calculated by the formula; [(TOS, μmol H2O2 equiv./L) / (TAS, mmol Trolox equiv./l) × 100] ^23^ .

MDA was measured with the spectrofluorometric method developed by Wasowicz *et al* . ^24^ . The method is based on the spectrofluorometric measurement of the fluorophore red product resulting from the reaction of MDA with thiobarbituric acid.

### Statistical analysis

Statistical Package for Social Sciences for Windows software (SPSS version 23.0, SPSS Inc., Chicago, Illinois, USA) was used for data analysis. The normal distributions of the variables were determined with Shapiro-Wilk's test. The variables without normal distribution were expressed as median (minimum-maximum) values and the variables with normal distribution were expressed as mean±standard deviation (SD). In case of normal distribution, the variables were compared with one-way ANOVA and in case of skewed distribution, Kruskal–Wallis test was used. Tukey's test and Dunn-Bonferroni pairwise comparison tests were used to compare the groups. A value of p<0.05 was accepted as statistically significant.

## Results

**Figure 2:**
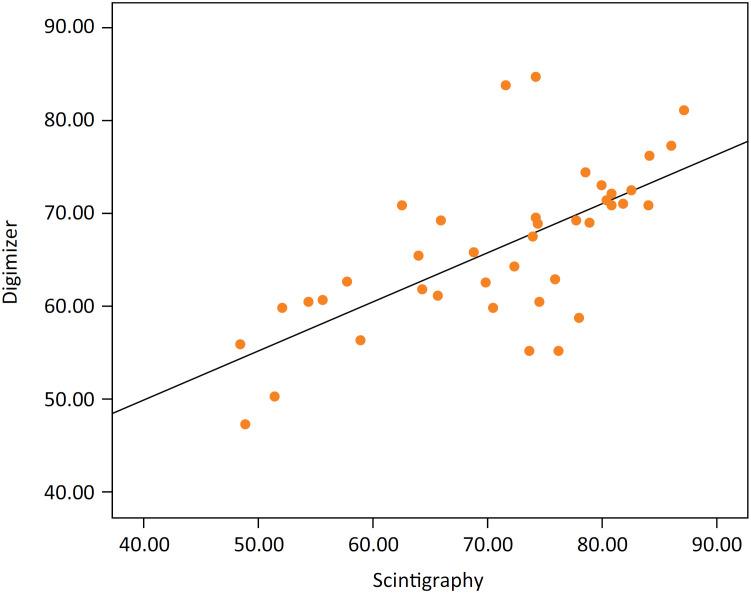
The correlation scatterplot showed a highly positive linear correlation between the photographic and the scintigraphic evaluation for viable flap area (r2 = 0.685, p=0.01).

All rats survived until the end of the study with no complications. Viable tissue percentages of scintigraphic and photographic analysis in all flap zones are presented in Table 1 . Perfusion analysis images were consistent with macroscopic flap images ( Fig. 1 ). The highest viable tissue percentages were found in Group DEX-CAL for both scintigraphic and photographic analysis ( Table 1 ). The differences between groups were significant in terms of the viable tissue percentages in both the scintigraphic evaluation and also in the photographic analysis, the values were as follows respectively for scintigraphic and photographic analysis, p<0.001, f=9.019 and p<0.001, f=14.625. Furthermore, scintigraphic analysis and photographic analyses were moderately correlated with each other for viable flap area (r ^2^ =0.685, p=0.01) ( Fig. 2 ).

**Table 1:** Viable tissue percentages of the groups in photographic analysis and in scintigraphy.

Viable tissue percentages in Groups	Photographic analysis	Scintigraphy
Group Sham	57.11 ± 5.2	57.88 ± 8.8
Group DEX	68.04 ± 7.5 *	74.08 ± 8.0 **
Group CAL	68.11 ± 7.3 *	72.82 ± 8.7 *
Group DEX-CAL	71.85 ± 6.7 **	79.19 ± 3.8 **
	a p < 0.001, f=9.019	a p < 0.001, f=14.625

a One-way ANOVA test has been used to compare groups,

* p< 0.01 when compared to Group Sham,

** p< 0.001 when compared to Group Sham.

The biochemical results show that TAS, TOS, OSI and MDA levels were significantly different between the groups. We found that Group DEX, Group CAL and Group DEX-CAL, TAS levels were significantly increased; however, TOS, OSI and MDA levels were significantly decreased compared to Group Sham ( Table 2 ). However, there was no significant difference between the treatment groups in terms of TAS, TOS, OSI and MDA levels.

**Table 2:** TAS, TOS, OSI and MDA levels of the groups.

	TAS	TOS	OSI	MDA
	mmol Trolox equiv./l	μmol H_2_O_2_ equiv./L		μmol/g
Group Sham	66.49 ± 28.9	10.48 ± 5.5	15.63±10.4	773.83 ± 253.0
Group DEX	133.94 ± 52.3 *	4.04 ± 1.4 ǂ	3.70±2.5 ǂ	359.95 ± 122.5 ǂ
Group CAL	122.05 ± 42.6 #	4.15 ± 2.4 ǂ	3.52±2.4 ǂ	376.21 ± 233.1 ǂ
Group DEX-CAL	115.60 ± 21.0 #	3.96 ± 1.9 ǂ	3.3±1.4 ǂ	400.72 ± 143.5 ǂ ǂ
	p < 0.001	p < 0.001 b	p < 0.001 b	p < 0.001 b
	f =6.044 a			

a One-way ANOVA and

b Kruskal-Wallis tests have been used to compare groups,

* p< 0.01 when compared to Group Sham,

# p< 0.05 when compared to Group Sham,

ǂ p< 0.01 when compared to Group Sham,

ǂ ǂ p< 0.05 when compared to Group Sham.

**Figure 3:**
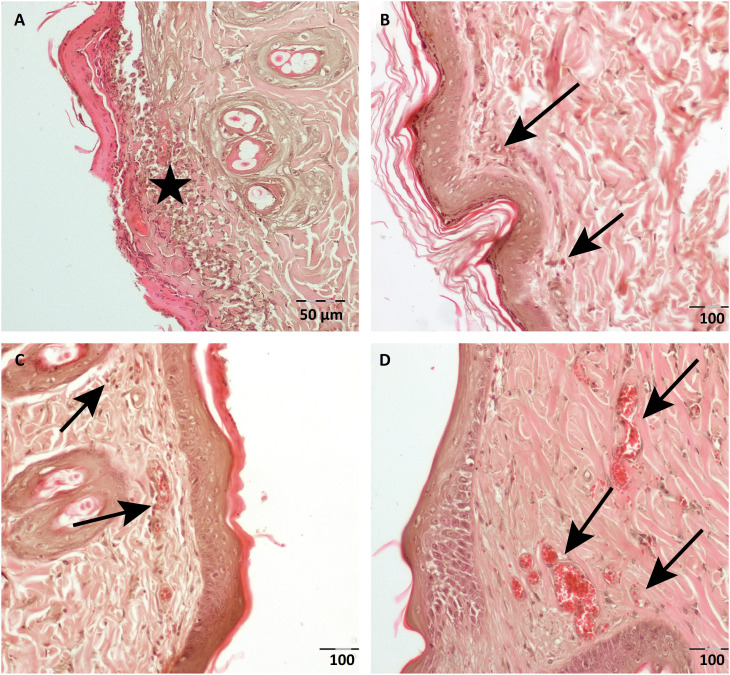
The representative photomicrographs of flap tissues are presented for **(a)** Group Sham, **(b)** Group DEX, **(c)** Group CAL and **(d)** Group DEX-CAL. The arrows are showing neovascularization, arrowheads are showing inflammation and stars are showing necrosis and inflammation.

In the histopathological evaluation, we found that the neovascularization values were significantly higher in Group DEX, Group CAL and Group DEX-CAL compared to Group Sham at the demarcation zone (p<0.001). On the other hand, the inflammation, edema and necrosis scores at demarcation zones were significantly higher in Group Sham compared to Group DEX, Group CAL and Group DEX-CAL (p values for inflammation, edema and necrosis scores respectively p<0.05, p<0.005, p<0.001) ( Table 3 ). However, no significant difference was found in terms of histopathological values between Group DEX, Group CAL and Group DEX-CAL ( Fig. 3 ).

**Table 3:** Histopathological results of the flap tissue expressed as mean ± standard deviation.

	Neovascularization		Inflammation		Edema		Necrosis	
Group Sham	19.5 ± 3.3	b p < 0.001	1.7 ± 1.1	b p < 0.05	2.7 ± 1.2	b p < 0.001	2.8 ± 1.0	b p < 0.001
Group DEX	26.8 ± 3.3 ǂ ǂ		1.2 ± 0.9 ǂ ǂ		1.2 ± 0.9 ǂ ǂ		1.2 ± 0.4	
Group CAL	26.6 ± 1.8		1.3 ± 0.7		1.2 ± 1.0		1.1 ± 0.6 ǂ ǂ	
Group DEX-CAL	31.0 ± 2.1		0.4 ± 0.7		0.8 ± 0.6		0.7 ± 0.7	

b Kruskal–Wallis test has been used to compare groups,

ǂ ǂ p<0.05 when compared to Group Sham.

## Discussion

The recovery of a random pattern skin flap remains as a main topic in the flap surgery. The pedicle of the flap restores its functions almost entirely; however, the challenging issue is the distal zone necrosis due to the insufficient blood flow and related IR injury. Therefore, to overcome IR injury with the least damage, several agents have been tried to increase the blood flow and reduce IR injury effects to random pattern skin flaps ^25^ ^-^ ^27^ .

After the flap surgery, as the size of the necrosis increases, there is more inflammation affecting the flap success and the weakening of inflammation accelerates healing when inflammatory responses are exacerbated ^28^ . Likewise, in our study, the edema, inflammation and necrosis were significantly higher in Group Sham compared to Group DEX, in Group CAL and Group DEX-CAL and correspondingly the flap viability percentages were significantly less in Group Sham compared to Group DEX, in Group CAL and Group DEX-CAL. This result confirmed that necrosis is increased by inflammation and also implicated the anti-inflammatory effects of Group DEX, in Group CAL and in Group DEX-CAL which are concordant with the anti-inflammatory properties of calcitriol, together with its ability to accelerate vascularization, suppress oxidative stress, and induce autophagy ^29^ and the anti-inflammatory effects of dexmedetomidine preconditioning ^9^ .

IR injury comprises an oxidation process, including the generation of reactive oxygen species (ROS) crucially affecting flap viability mainly at the time of reperfusion ^30^ . At the beginning of oxidative stress, ROS react with the cell and mitochondrial membranes lipids and proteins, activating peroxidation and eventually causing the flap necrosis by destroying cells and tissues. Tissue TAS and TOS reflect the redox balance between oxidation and antioxidation and MDA levels, as a marker of lipid peroxidation reflects the extent of tissue injury. Through the literature, MDA level increase has been already shown in after flap IR injury rat models ^31^ ^,^ ^32^ . Similarly, we found that our random skin flap model induces significant increases in MDA and TOS levels and reduces TAS levels as a result of IR in Group Sham.

Moreover, it has been shown that calcitriol use and dexmedetomidine use reduce tissue oxidant parameters and increased anti-oxidant parameters when used separately. Such as an IR injury study in rat hippocampus showed that calcitriol decreases MDA levels compared to the control group and protects from IR injury ^33^ .

A heart IR study ^5^ and also a study evaluating the testis IR demonstrated that dexmedetomidine significantly increased TAS, and significantly decreased OSI in testis tissue ^7^ . Furthermore, an epigastric island flap study evaluating dexmedetomidine preconditioning showed that dexmedetomidine shows antioxidant effects by decreasing ROS and by inhibiting lipid peroxidation and therefore decreases IR injury and improves viability after IR injury compared to Sham group ^9^ . Similarly, a random skin flap rat model evaluating the effects of calcitriol showed anti-inflammatory effects with decreased MDA levels in groups preconditioned with calcitriol ^18^ . Likewise, we found that both dexmedetomidine and calcitriol and also their combination as a preconditioning treatment significantly increased TAS and significantly decreased TOS, OSI and MDA levels, confirming the antioxidant effects in Group DEX, Group CAL and Group DEX-CAL. However, though the mean TAS levels were highest in Group DEX-CAL and TOS and MDA levels were lowest in Group DEX-CAL, we found no significant statistical difference of TAS, TOS and MDA levels between Group DEX, Group CAL and Group DEX-CAL.

The results of a study in an IR injury in rats, found that post-surgical treatment with dexmedetomidine may increase the expression of VEGF ^34^ and also studies showing that calcitriol increases VEGF expression and release in vascular smooth muscle cells ^35^ ^,^ ^36^ , which may have the role in increasing flap viability and support our findings. However, in our study, we have evaluated the oxidative mechanisms and assessed the angiogenesis morphologically in flap viability other than focusing on neovascular angiogenesis, tissue regeneration, or hypoxia with specific biomarkers such as VEGF, transforming growth factor beta (TGF-beta), and hypoxia inducible factor 1 (HIF-1); we think that studying these biomarkers definitely warrants a further study to confirm our results.

The histopathological assessment of our study revealed that the inflammation, edema and necrosis scores diminished and neovascularization increased in Group DEX, Group CAL and Group DEX-CAL compared to the Group Sham. The mean inflammation, edema and necrosis scores were lowest in Group DEX-CAL, and highest in Group Sham.

Furthermore, we have confirmed our findings tissue oxidant/antioxidant parameters and histopathologic assessment with scintigraphic analysis and also with photographic analysis. And to our knowledge, this is the first study evaluating the flap viability by biochemical, scintigraphic, photographic and histopathologic analysis. We found that, concordant with the histopathological results and tissue oxidant/antioxidant parameters we found that the viability of the flap increases in Group DEX, Group CAL and Group DEX-CAL both by scintigraphic evaluation and also by photographic analysis. Similarly, the highest viability of the flap was in Group DEX-CAL and lowest viability of the flap was in Group Sham.

Our results show that preconditioning with both dexmedetomidine and calcitriol and also their combination decreased ROS and reduced IR injury and therefore by this mechanism significantly improved the random flap viability. Due to different mechanisms of action of dexmedetomidine and calcitriol in preventing the IR injury, the combination of both treatment in Group DEX-CAL has shown better scintigraphic, photographic, histopathologic analysis and tissue oxidant/antioxidant parameters confirmed our results.

## Conclusion

The combination of dexmedetomidine and calcitriol warrants protective effect on flap viability that should be considered in patients for planned flap surgery.
