# ERRATUM

**DOI:** 10.1590/s0102-865020200090000003err

**Published:** 2020-10-28

**Manuscript:** The outcomes of dexmedetomidine and calcitriol on flap viability

**Publication:** Acta Cir Bras. 2020;35(9):e202000903.

**DOI:** http://dx.doi.org/10.1590/s0102-865020200090000003

On the Title Page of the original publication, instead:

^IX^ MD, PhD, Department of Anesthesiology and Reanimation, University of Health Sciences, Ankara Training and Application Hospital, Ankara, Turkey. Critical revision, final approval.

Consider this:

^IX^ MD, PhD, Department of Nuclear Medicine, University of Health Sciences, Ankara Training and Application Hospital, Ankara, Turkey. Critical revision, final approval.

